# The *Frizzled 3 *gene is associated with methamphetamine psychosis in the Japanese population

**DOI:** 10.1186/1744-9081-4-37

**Published:** 2008-08-15

**Authors:** Makiko Kishimoto, Hiroshi Ujike, Yuko Okahisa, Tatsuya Kotaka, Manabu Takaki, Masafumi Kodama, Toshiya Inada, Mitsuhiko Yamada, Naohisa Uchimura, Nakao Iwata, Ichiro Sora, Masaomi Iyo, Norio Ozaki, Shigetoshi Kuroda

**Affiliations:** 1 Department of Neuropsychiatry, Okayama University Graduate School of Medicine, Dentistry and Pharmaceutical Sciences, Okayama, Japan; 2 JGIDA (Japanese Genetics Initiative for Drug Abuse), Japan; 3 Institute of Neuropsychiatry, Seiwa Hospital, Tokyo, Japan; 4 Department of Psychogeriatrics, National Institute of Mental Health, National Center of Neurology and Psychiatry, Kodaira, Japan; 5 Department of Neuropsychiatry, Kurume University Graduate School of Medicine, Kurume, Japan; 6 Department of Psychiatry, Fujita Health University School of Medicine, Houmei, Japan; 7 Department of Neuroscience, Division of Psychobiology, Tohoku University Graduate School of Medicine, Sendai, Japan; 8 Department of Psychiatry, Chiba University Graduate School of Medicine, Chiba, Japan; 9 Department of Psychiatry, Nagoya University Graduate School of Medicine, Nagoya, Japan

## Abstract

**Background:**

Frizzled 3 (Fzd3) is a receptor required for the Wnt-signaling pathway, which has been implicated in the development of the central nervous system, including synaptogenesis and structural plasticity. We previously found a significant association between the *FZD3 *gene and susceptibility to schizophrenia, but subsequent studies showed inconsistent findings. To understand the roles of the *FZD3 *gene in psychotic disorders further, it should be useful to examine *FZD3 *in patients with methamphetamine psychosis because the clinical features of methamphetamine psychosis are similar to those of schizophrenia.

**Methods:**

Six SNPs of *FZD3*, rs3757888 in the 3' flanking region, rs960914 in the intron 3, rs2241802, a synonymous SNP in the exon5, rs2323019 and rs352203 in the intron 5, and rs880481 in the intron 7, were selected based on the previous schizophrenic studies and analyzed in 188 patients with methamphetamine psychosis and 240 age- and gender-matched controls.

**Results:**

A case-control association analyses revealed that two kinds of *FZD3 *haplotypes showed strong associations with methamphetamine psychosis (*p *< 0.00001). Having the G-A-T-G or A-G-C-A haplotype of rs2241802-rs2323019-rs352203-rs880481 was a potent negative risk factor (odds ratios were 0.13 and 0.086, respectively) for methamphetamine psychosis.

**Conclusion:**

Our present and previous findings indicate that genetic variants of the *FZD3 *gene affect susceptibility to two analogous but distinct dopamine-related psychoses, endogenous and substance-induced psychosis.

## Background

The neurodevelopmental hypothesis of schizophrenia suggests that interaction between genetic and environmental events occurring during critical early periods of neuronal growth may negatively influence the way by which nerve cells are laid down, differentiated, selectively culled by apoptosis and remodeled by expansion and retraction of dendrites and synaptic connections [1,2]. The Wnt family molecules play several roles in neuronal development by inducing cells to proliferate, differentiate, and survive [3,4]. In particular, Wnt signaling plays roles in regulating patterning during cortical development, axon remodeling, synaptic differentiation, clustering of synapsin I at presynaptic terminals [5-7] and the cytoarchitectural derangement that was observed in the brains of schizophrenics [8]. A mutation in the Wnt1 gene, one of the Wnt family genes, leads to abnormal cerebral development in mice [9], and mice deficient in Frizzled 3 (Fzd3), a receptor of Wnt ligands, showed loss of thalamo-cortical tracts and defects in corpus callosum development, abnormalities which were reported in schizophrenic patients [10-12]. Therefore, alteration of the Wnt/Fzd cascade may represent an aberrant neurodevelopment involved in schizophrenia [13].

Fzd3 is a required receptor in the Wnt-signaling pathway. In 2003, we reported a significant association between the gene encoding Fzd3 (*FZD3) *and susceptibility to schizophrenia [14]. Subsequent studies tried to replicate our findings, but the results were inconsistent. Yang et al. [15] revealed a significant association of the *FZD3 *gene with schizophrenia in Han Chinese populations by a transmission disequilibrium test, and Zhang et al. [16] also found a significant association by a family-based case-control study. On the other hand, several studies failed to find significant evidence of a genetic effect of the *FZD3 *gene on schizophrenia [17-19]. The inconsistencies in genetic studies in the relationship of the *FZD3 *gene with schizophrenia may suggest heterogeneity of schizophrenia and a requirement for further studies using larger sample size. We consider that it may be also useful to investigate the role of the *FZD3 *gene in other types of psychotic disorders for better understanding of the physiological roles of Fzd3 and the Wnt cascade in schizophrenia or psychotic conditions.

Repeated abuse of methamphetamine frequently predisposes to psychotic conditions. The clinical similarity between methamphetamine psychosis and schizophrenia has been pointed out, and methamphetamine psychosis has been considered to be a pharmacological model of schizophrenia, especially the paranoid subtype [20-22]. Thus, methamphetamine psychosis and schizophrenia resemble each other in a cross-section of clinical features, e.g., auditory hallucination and delusion, the longitudinal process of progressive exacerbation with acute relapses, good response to neuroleptics, and enduring vulnerability to relapse under stressors. Enhanced dopamine release in the striatum due to a challenge dose of methamphetamine was observed in schizophrenic patients and methamphetamine-sensitized rats, an animal model of methamphetamine psychosis [23-25]. These similarities between schizophrenia and methamphetamine psychosis in both symptomatology and pharmacological aspects may suggest that shared neural mechanisms are involved in both psychotic disorders. Therefore, in order to examine the roles of Fzd3 in mechanisms underlying the development of psychosis, we analyzed the *FZD3 *gene in patients with methamphetamine psychosis.

## Methods

### Subjects

The subjects consisted of 188 patients with methamphetamine psychosis (158 male, 30 female; mean age ± SD, 36.6 ± 11.8) and 240 age-, gender-, and geographical origin-matched healthy controls (192 male, 48 female; mean age ± SD, 36.6 ± 10.6), who have no individual or family history of drug dependence or major psychotic disorders such as schizophrenia and bipolar disorders. All the subjects were unrelated Japanese, born and living in relatively restricted areas of Japan, northern Kyushu, Setouchi, Chukyo, Tokai, and Kanto. All subjects were out-patients or inpatients in psychiatric hospitals of the Japanese Genetics Initiative for Drug Abuse (JGIDA), a multicenter collaborative study group. Consensus diagnoses of methamphetamine psychosis were made by two trained psychiatrists according to the ICD-10 criteria on the basis of interviews and medical records. The patients with methamphetamine psychosis in the present study usually showed predominant positive symptoms such as delusion and hallucination. We excluded cases in which the predominant symptoms were of the negative and/or disorganized type in order to maintain the homogeneity of the patient group. The study protocol and purpose were explained to all subjects participating in the study, and written informed consent was obtained from all subjects. This study was approved by the Ethics Committee of each participating institute of JGIDA.

### DNA analysis

We genotyped the three single nucleotide polymorphisms (SNPs), rs3757888 (SNP1) in the 3' flanking region, rs960914 (SNP2) in the intron 3, and rs2241802 (SNP3), a synonymous SNP in the exon5 of the *FZD3 *gene that were analyzed in our previous study [14]. We also analyzed three additional SNPs, rs2323019 (SNP4) and rs352203 (SNP5) in the intron 5, and rs880481 (SNP6) in the intron 7 of the gene because a significant association with schizophrenia was reported by Yang et al. [15] and Zhang et al. [16]. Genotyping was performed by the PCR-RFLP method. The genomic DNA was extracted from peripheral leukocytes using phenol-chloroform. Each polymorphic site was amplified by PCR (PCR primer sequence of each SNP is available on request) in a 15-μl volume containing 3% dimethyl sulfoxide and 0.75 units of Taq DNA polymerase (Promega Co., Japan) using a unique primer set. PCR reaction was performed under the following conditions: 95°C for 5 min, then 35 cycles of 30 s of denaturing at 95°C, 1 min of annealing at the appropriate temperature, and 30 s of extension, and final elongation at 72°C for 10 min. The PCR products were digested with the corresponding restriction enzyme for each polymorphism, Dde*I *for rs3757888, Rsa*I *for rs960914, Alu*I *for rs2241802, Ssp*I *for rs2323019, Nla*III *for rs352203, Eco32*I *for rs880481, and then electrophoresed on 3.0% agarose gels and stained with GelStar (TaKaRa Co., Japan). All genotyping was performed in a blinded fashion, with the control and cases samples mixed randomly. The genotyping of the SNPs were confirmed in part by direct sequencing or a TaqMan SNP genotyping assay (Applied Biosystems, Foster City, CA, U.SA.).

### Statistical analysis

Statistical analysis of association was performed using SNPAlyze software (Dynacom Co., Japan). Deviation from Hardy-Weinberg equilibrium and case- control study were tested using the χ^2 ^test for goodness of fit and χ^2 ^test for dependence, respectively. Linkage disequilibrium (LD) was tested using the χ^2 ^test, and D' and r^2 ^values were made the index in the authorization of LD. Case-control haplotype analysis was performed by the permutation method, and permutation *p*-values were calculated based on 100,000 replications.

## Results

The genotype distribution and allele frequencies of the each polymorphism are shown in Table 1. The genotype distributions of patients and control subjects did not deviate from Hardy-Weinberg equilibrium at any SNP examined. The allele frequencies of SNP1, SNP2, and SNP3 were approximately same as those of our previous study [14]. The allele frequencies of SNP4, SNP5, and SNP6 in the present study also showed values similar to those of previous studies of Japanese and Chinese populations [16-18].

**Table 1:** Genotype and allele distribution of six SNPs of the FZD3 gene in controls and patients with methamphetamine (MAP) psychosis

		**Genotype**				***p***	**Allele**		***p***
		
SNP1	rs3757888	N	A/A	A/G	G/G		A	G	
Control		230	198(86.1)	31(13.5)	1(0.4)		427(92.8)	33(7.2)	
MAP Psychosis		186	151(81.2)	32(7.2)	3(1.61)	0.26	334(89.8)	38(10.2)	0.19
									
SNP2	rs960914	N	T/T	T/C	C/C		T	C	

Control		240	67(27.9)	130(54.2)	43(17.9)		264(55.0)	216(45.0)	
MAP Psychosis		185	45(24.3)	103(55.7)	37(20.0)	0.66	193(52.2)	177(47.8)	0.41
									
SNP3	rs2241802	N	A/A	A/G	G/G		A	G	

Control		240	49(20.4)	124(51.7)	67(27.9)		222(46.2)	258(53.8)	
MAP Psychosis		181	44(24.3)	97(53.6)	40(22.1)	0.34	185(51.1)	177(48.9)	0.16
									
SNP4	rs2323019	N	A/A	A/G	G/G		A	G	

Control		239	72(31.4)	113(49.3)	44(19.2)		257(56.1)	201(43.9)	
MAP Psychosis		186	45(24.1)	101(54.0)	41(21.9)	0.25	191((51.1)	183(48.9)	0.15
									
SNP5	rs352203	N	T/T	T/C	C/C		T	C	

Control		192	64(33.3)	98(51.1)	30(15.6)		226(58.9)	158(41.1)	
MAP Psychosis		176	49(27.8)	98(55.7)	29(16.5)	0.52	196(55.7)	156(44.3)	0.38
									
SNP6	rs880481	N	A/A	A/G	G/G		A	G	

Control		236	43(18.2)	123(52.1)	70(29.7)		209(44.3)	263(55.7)	
MAP Psychosis		186	30(16.1)	103(55.4)	53(28.5)	0.97	163(43.8)	209(56.2)	0.99

SNP, Single nucleotide polymorphism.

Numbers in parentheses indicate percentages.

We found no significant difference between patients and controls in the frequencies of the genotype or allele at any single SNP of the *FZD3 *gene. We estimated the pairwise LD between the six SNPs of the *FZD3 *gene using the D' and r^2^values as an index (Table 2). A D' range of 0.7–0.9 and a r^2 ^> 0.3 were found between SNP2, SNP3, SNP4, SNP5, and SNP6, but not between SNP1 and the other SNPs. This suggests that SNP2, SNP3, SNP4, SNP5, and SNP6 are in linkage disequilibrium and located within one LD block. Then, we performed case-control haplotype analysis using SNP2 to SNP6 (Table 3). Haplotype analyses revealed significant differences in patients and control subjects at SNP5-6, SNP4-5-6, SNP3-4-5-6, and SNP2-3-4-5-6, but not at SNP2-3, SNP3-4, SNP4-5, SNP2-3-4, SNP3-4-5, or SNP2-3-4-5. The largest χ^2 ^and smallest permutation *P *values were found in the haplotype analysis of SNP3-4-5-6 (χ^2 ^= 64.8, permutation *p *< 0.00001). The estimated individual haplotypic frequencies of SNP3-4-5-6 are shown in Table 4. Eight kinds of haplotypes consisting of SNP3-4-5-6 with more than 1% overall frequency were identified. The estimated haplotype frequency of G-A-T-G and A-G-C-A of SNP3-4-5-6 were significantly lower in patients with methamphetamine psychosis than in controls (*p *< 0.00001 and *p *= 0.0003, respectively). Conversely, the A-G-C-G haplotype was significantly in excess in patients compared with controls (*p *= 0.0246). To avoid a type I error due to multiple comparison, Bonferroni's correction was applied to the results. G-A-T-G and A-G-C-A haplotypes were still significantly less frequent in the methamphetamine patients than in the controls, but A-G-C-G was not significantly different between the groups after correction. The odds ratios G-A-T-G and A-G-C-A haplotypes were 0.13 (95%CI; 0.043–0.36) and 0.086 (95%CI; 0.011–0.67), respectively. Accordingly, G-A-T-G and A-G-C-A haplotypes of SNP3-4-5-6 were negative risk haplotypes for methamphetamine psychosis.

**Table 2:** Pairwise Linkage Disequilibrium between six SNPs of the FZD3 gene

	SNP1	SNP2	SNP3	SNP4	SNP5	SNP6
SNP1		**0.840**	0.557	0.379	**0.853**	**0.706**
SNP2	0.057		**0.760**	**0.915**	**0.970**	**0.749**
SNP3	0.031	**0.532**		**0.834**	**0.831**	**0.729**
SNP4	0.012	**0.829**	**0.627**		**0.982**	**0.760**
SNP5	0.052	**0.841**	**0.542**	**0.843**		**0.788**
SNP6	0.036	**0.377**	**0.389**	**0.387**	**0.367**	

Linkage disequilibrium was tested using χ^2 ^test. Upper right and lower left diagonals show D' and r-square values, respectively. D'>0.7 and r-square>0.3 were shown in bold.

**Table 3:** Haplotype analysis of the FZD3 gene

	1SNP	2SNP	3SNP	4SNP	5SNP
	
SNP ID			Permutation p-value
SNP2	0.41				
(rs960914T>C)		0.16			
SNP3	0.16		0.22		
(rs2241802G>A)		0.15		0.35	
SNP4	0.15		0.15		**<0.00001**
(rs2323019A>G)		0.072		**<0.00001**	
SNP5	0.38		**0.00001**		
(rs352203T>C)		**0.00002**			
SNP6	0.99				
(rs880481A>G)					

Haplotype analysis was performed by permutaion method. Bold values represent significant p values.

**Table 4:** Haplotype frequencies from positive permutation analyses

Haplotype	Frequency		Permutation p-values	Odds ratio (95%CI)
		
(SNP3-4-5-6)	Controls	MAP Psychosis		
G-A-T-A	0.3523	0.4148	0.0889	
A-G-C-G	0.3178	0.3970	0.0246	1.42 (1.14–1.76)
G-A-T-G	0.1542	0.0243	<0.00001	0.13 (0.07–0.22)
A-A-T-G	0.0382	0.0635	0.1283	
A-G-C-A	0.0625	0.0070	0.0003	0.086 (0.03–0.24)
A-G-T-G	0.0211	0.0354	0.2791	
G-G-C-G	0.0196	0.0379	0.1678	
A-A-T-A	0.0169	0.0090	0.4565	

Haplotypes with overall frequencies are less than 1% were eliminated.

## Discussion

We revealed that the *FZD3 *gene is significantly associated with the vulnerability to psychosis induced by methamphetamine abuse, and two haplotypes of the *FZD3 *gene comprising SNP3-4-5-6 (rs2241802-rs2323019-rs352203-rs880481) were identified as potent negative risk factors for methamphetamine psychosis. The G-A-T-G and A-G-C-A haplotypes potently reduce the risks of predisposition to psychosis after methamphetamine abuse to one seventh to one eleventh. In our previous study of schizophrenia [14], distribution of the SNP2 genotypes and haplotypes comprising SNP2-SNP3 was significantly associated with schizophrenia. Zhang et al. [16] reported that the haplotype comprising SNP4-SNP5-SNP6 was associated with schizophrenia in a Chinese population. These findings indicate that genetic variants of the *FZD3 *gene may affect susceptibility to two analogous but distinct psychoses, endogenous psychosis of schizophrenia and substance-induced psychosis. This may imply that Fzd3 is involved in a liability to psychotic symptoms such as hallucination and delusion irrespective of whether they are due to schizophrenia or methamphetamine psychosis.

Dopamine is a key molecule in the pathophysiology of both schizophrenia and methamphetamine psychosis. Enhanced dopamine release in the terminals of mesolimbic dopamine projections was demonstrated *in vivo *in patients with schizophrenia, and the amount of the increase in dopamine was positively associated with the emergence or worsening of psychotic symptoms [25]. Similar phenomena were demonstrated in mesolimbic and mesocortical terminals in animal models of methamphetamine psychosis [23]. Wnt1 was found to be expressed in close vicinity to developing midbrain dopamine neurons, which are the origins of the mesolimbic and mesocortical dopamine pathways. Wnt1 regulates the genetic network leading to establishment of the midbrain progenitor domain in the ventral midbrain during embryonic development and of the subsequent terminal differentiation of midbrain dopamine neurons [26,27]. It is possible that differences in Wnt signaling due to genetic variants of the *FZD3 *gene affect the development of dopamine neurons of the mesolimbic or mesocortical pathway in early brain development and susceptibility to these two dopamine-related psychoses in adulthood.

Another molecule that potentially links Fzd3 and these two related psychoses is glycogen synthesis kinase-3 (GSK-3), a serine/threonine kinase that is a downstream component of the Wnt/Fzd cascades. Binding of Wnt ligands to Fzd family receptors leads to activation of the intracellular protein disheveled, which inactivates GSK-3β. This in turn leads to the stabilization and accumulation of β-catenin, which translocates to the nucleus where it interacts with nuclear transcription factors for the genes involved in neuronal development. Briefly, GSK-3β mediates Wnt/Fzd signaling cascades. Dysregulation of GSK-3β and 3α is one of promising neurodevelopmental hypotheses of schizophrenia [13,28]. GSK-3 is also regulated by dopamine signaling through protein kinase B [29]. Several studies showed, but not consistently, that GSK-3 protein levels and activities are altered in schizophrenic brains [30,31] and lymphocytes [32,33]. Several genes, e.g., *DISC1 *and *NRG1*, which have been repeatedly shown to be associated with susceptibility to schizophrenia, are involved in GSK-3/Wnt regulatory pathways [28]. Recently, the gene encoding DKK4, a component of the GSK-3/Wnt signaling cascade, was shown to be associated with schizophrenia. DKK4 inhibits Wnt-Fzd binding, resulting in inactivation of GSK-3 [34]. On the other hand, amphetamine also affects GSK-3 activity. Administration of amphetamine to mice increased Ser9 phosphorylation of GSK-3β, resulting in a reduction of its activity in the frontal cortex and striatum [35], and GSK-3 gene knockdown mice showed a reduced response to amphetamine [36]. Intriguingly, psychotomimetics of two different classes, phencyclidine and D-lysergic acid, also had the same effects on GSK-3β, which may imply that substance-induced psychosis might be the result of a reduction in GSK-3 signaling. In contrast, chronic treatment with typical and atypical neuroleptics that ameliorate the psychotic symptoms of schizophrenia and methamphetamine psychosis increase the levels and activities of GSK-3 [37]. It was also found that chronic neuroleptic treatment increased β-catenin in the ventral midbrain, whereas amphetamine decreased it [38]. These findings indicate that the altered GSK-3/Wnt signaling is involved in liability to expression of positive psychotic symptoms such as the hallucinations and delusions in patients suffering from both schizophrenia and methamphetamine-induced psychosis. This hypothesis may be supported by our present and previous findings because the *FZD3 *gene was significantly associated with not only schizophrenia but also methamphetamine psychosis.

The present results were still significant even after a Bonferroni correction, although it is possibly a chance finding due to less power. The power analysis showed that our present sample size had more than 80% power to detect a significant difference at 0.05 of any SNP examined, but it must have less power for haplotype analyses. Therefore, our findings should be confirmed in studies using a larger number of subjects and different populations. It may also be useful for further investigation of the roles of Fzd3 in psychoses to examine the genetic association of the *FZD3 *gene with other types of psychoses, e.g., cocaine-induced paranoia or delusional type of bipolar disorders.

## Conclusion

We examined genetic association of *FZD3 *and found that two kinds of *FZD3 *haplotypes showed strong associations with methamphetamine psychosis. Having the G-A-T-G or A-G-C-A haplotype of rs2241802-rs2323019-rs352203-rs880481 was a potent negative risk factor (odds ratios were 0.13 (95%CI; 0.07–0.22) and 0.086 (0.03–0.24), respectively) for methamphetamine psychosis. Our present and previous findings indicate that genetic variants of the *FZD3 *gene affect susceptibility to two analogous but distinct dopamine-related psychoses, endogenous and substance-induced psychosis.

## Competing interests

The authors declare that they have no competing interests.

## Authors' contributions

HU conceived of the study, reviewed the manuscript and supervised all management, analysis, and interpretation of the data. MKi, YO, TK supervised by MT and MKo, genotyped samples and analyzed data, and MKi drafted manuscript and produced all tables. HU organized collaboration of Japanese substance abuse group, and HU, TI, MY, NU, NI, IS and NO collected genome samples and informed consents. HU and SK managed research expense. All authors read and approved for final manuscript.
